# Study on the Chlorine Resistance of HDPE Pipe to Chlorinated Seawater

**DOI:** 10.3390/polym18202572

**Published:** 2026-10-19

**Authors:** Chao Cheng, Yongjing Li, Jinpeng Xiao, Huan Sheng Lai, Fangjie Shi

**Affiliations:** 1 China Nuclear Power Engineering Co., Ltd., Shengzhen 518124, China; 2 Sino-French Institute of Nuclear Engineering and Technology, Sun Yat-Sen University, Zhuhai 519082, China; 3 Suzhou Nuclear Power Research Institute Co., Ltd., Suzhou 215004, China

**Keywords:** HDPE pipe, chlorinated seawater, chlorine-induced aging, brittle fracture

## Abstract

High-density polyethylene (HDPE) pipes are widely used for seawater transportation. However, residual chlorine introduced for disinfection purposes can induce oxidative degradation and accelerate material failure. Existing investigations predominantly focus on HDPE aging in chlorinated freshwater, while the degradation mechanism of HDPE subjected to chlorinated seawater remains poorly documented. In this paper, chlorine-resistance tests for HDPE pipes were performed following the ASTM F2263 standard at 90 °C using real seawater with 0.2 ppm free residual chlorine. Micro-morphology, elemental distribution, and tensile properties were characterized by scanning electron microscopy (SEM), energy-dispersive spectroscopy (EDS), and uniaxial tensile tests. The chlorine-resistance test results revealed that HDPE pipes eventually failed in a brittle-fracture mode after long-term exposure to chlorinated seawater. Numerous axial surface-initiated microcracks formed on the pipe inner wall, while EDS line scan demonstrated that chloride ions hardly diffused into the bulk polymer matrix. Reducing hoop stress by 18% only extended failure lifetime by approximately 6%, indicating a weak linear correlation between hoop stress and failure time under the present chemo-mechanical coupling condition. This study provides experimental references for the safety evaluation of HDPE pipes serving in chlorinated seawater environments.

## 1. Introduction

Compared with metallic piping systems, high-density polyethylene (HDPE) pipes feature low manufacturing, transportation, operation and maintenance costs coupled with excellent corrosion resistance, making them widely applied for transporting potable water and seawater [1,2,3]. Sodium hypochlorite (NaClO) or chlorine dioxide (ClO_2_) is commonly dosed into water to inactivate microorganisms for disinfection and mitigate pipeline blockages induced by microbiologically influenced corrosion [4,5]. However, the strong oxidizing capacity of hypochlorite species can trigger corrosive oxidative aging of HDPE pipes and substantially curtail their service lifetime [5,6,7,8,9]. Consequently, it is of practical significance to explore the chlorine-driven corrosive aging mechanisms of HDPE pipes.

Considerable experimental efforts have been devoted to understanding the oxidative aging performance of HDPE pipes [10]. Hot-water aging tests demonstrate that oxidative degradation of HDPE pipes initiates upon the depletion of antioxidants [11]. Mutual chemical interactions among oxidants, antioxidants and polymer matrices consume antioxidant additives and alter the chemical structure of polyethylene. The hydrolysis, leaching, diffusion and intrinsic chemical stability of antioxidants are recognized as critical factors governing the long-term service performance of HDPE pipes [12]. Chlorine-tolerant antioxidants are therefore compounded into pipe grades to enhance anti-chlorine-aging performance. Once antioxidants are exhausted, the inner layer of HDPE pipes undergoes degradation accompanied by microcrack nucleation; the crack initiation rate depends jointly on material degradation kinetics and local stress intensity [13]. Stress intensity prevails as the dominant factor under high-stress conditions, while degradation kinetics governs failure under low-stress yet high-chlorine-oxidation environments. The interplay between chemical degradation and mechanical stress renders the overall failure mechanism sensitive to material formulation, chlorinated medium chemistry and applied stress magnitude. It has also been revealed that sodium hypochlorite penetrates the amorphous domains of polyethylene, generating free-radical intermediates and initiating radical-mediated oxidation reactions, which ultimately degrade mechanical performance and shorten service lifetime [14].

Generally, accelerated aging evaluations for polyethylene pipes are performed by immersing specimens in ClO_2_- or NaClO-containing aqueous solutions [15,16]. To enable realistic assessment of HDPE pipe performance under in-service conditions, the American Society for Testing and Materials (ASTM) has released the ASTM F2263 standard to specify the chlorine-resistance test of the HDPE pipe [17]. This type of chlorine-resistance test has been adopted by several authors [4,7,18]. Furthermore, numerical and analytical models have been established to characterize the chlorine-induced aging evolution of polyethylene materials [8,19,20,21].

Despite abundant existing achievements regarding the chlorine-oxidation resistance of HDPE pipes, most published experiments are implemented in freshwater systems with residual chlorine. Specimens implemented in freshwater normally fail under the combined action of tensile stress and residual chlorine oxidation, which differs substantially from the failure mechanism of specimens in the chlorine-resistance test following ASTM F2263 [17]. In the chlorine-resistance test, failure occurs in pressurized flowing hot chlorinated water under the combined effects of hoop stress, axial stress, and residual chlorine oxidation. Furthermore, little attention has been paid to water-quality effects on HDPE aging behavior. Specifically, the degradation response of HDPE pipes exposed to chlorinated seawater remains poorly characterized.

In this study, the chlorine-induced aging behavior of HDPE pipes in seawater containing residual chlorine was investigated in accordance with the ASTM F2263 standard test [17]. Chlorine-resistance tests were carried out in seawater at 90 °C with 0.2 ppm free residual chlorine. Post-test microstructural evolution and elemental distribution were characterized by scanning electron microscopy (SEM) coupled with energy-dispersive spectroscopy (EDS). Changes in mechanical performance were further assessed through tensile tests.

## 2. Experiment

Chlorine-resistance tests were carried out in accordance with the ASTM F2263 standard test [17] in this study. The chlorine-resistance test was performed using a CLO-115 chlorine-resistance tester (Guangdong Jundeyu Technology Co., Ltd., Guangzhou, China). Pressurized hot chlorinated fluid circulated through the interior of pipe specimens, while the external surface of each specimen was exposed to an air environment held at identical temperature throughout the test. The instrument provided automatic closed-loop control over test pressure and temperature, with accuracies of ±0.015 MPa and ±1 °C, respectively. NaClO was dosed into the circulating fluid to adjust the free-chlorine concentration, and acetic acid (CH_3_COOH) was added for pH regulation. The pH value and free-chlorine concentration were automatically maintained with control accuracies of ±0.1 and ±0.2 ppm, correspondingly. Hence, the testing apparatus complied with the requirements specified in the ASTM F2263 standard [17].

The pipe material used in this study was HDPE. It was an IPS (iron-pipe-size) 1/2 in. pipe with a dimension ratio of 11, corresponding to an outer diameter of 21.34 mm and a wall thickness of 1.94 mm. Pipe segments with a total length of 400 mm were machined as chlorine-resistance test specimens, as shown in Figure 1a. Therefore, the specimen was prepared in full accordance with the ASTM F2263 standard [17]. The experimental water was seawater, which was taken from the offshore area of Tangjiawan Town, Zhuhai City, Guangdong Province, China. The raw seawater was sequentially pre-filtered through GF/F glass fiber filters and finely filtered using 0.45 μm PES (polyethersulfone) membrane filters, and subsequently diluted 100-fold with pure water to prepare the seawater sample for composition analysis, owing to the excessively high concentrations of chloride and other ions. An ion chromatograph (Thermo Scientific Dionex ICS-5000, Thermo Fisher Scientific, Waltham, MA, USA) was employed to determine the anion composition, while another ion chromatograph (ICS-900, Thermo Fisher Scientific, Waltham, MA, USA) was used for cation measurement. The measured composition of seawater is listed in Table 1. In addition, seawater composition varies among different marine regions and may affect the experimental results. Therefore, further work is required to investigate the influence of seawater composition on experimental results in future studies.

According to the ASTM F2263 standard [17], two specimens comprised a unit, which was tested at the same temperature and inner pressure. Two units were tested at the same temperature of 90 °C and at two different inner pressures of 0.8 MPa and 0.66 MPa until specimen failure. The internal pressures of 0.8 MPa and 0.66 MPa induced hoop stresses of 4.0 MPa and 3.3 MPa, respectively, as listed in Table 2. The difference between the two hoop stresses was 0.7 MPa, exceeding the minimum required value of 0.55 MPa specified in ASTM F2263 [17]. The pH and free residual chlorine were set as 6.9 and 0.2 ppm during the test. Since seawater was used, the oxidation reduction potential (ORP) was 612 mv under the set values of pH and free residual chlorine.

In addition, tensile tests were performed on both HDPE pipes without and after chlorine-resistance test. The geometry of the tensile specimens is shown in Figure 1b. SEM coupled with EDS was used to characterize the microstructural features of the specimens as well as elemental distribution across the wall thickness.

## 3. Results and Discussion

All specimens exhibited brittle-mode failure after testing durations exceeding 2000 h, and each failure originated from a single brittle crack. The representative brittle-failure morphology of HDPE pipe specimens subjected to chlorine-resistance testing at 90 °C is shown in Figure 2. Macroscopically, a brittle crack was observed on the specimen surface (Figure 2a), without obvious large-scale plastic deformation prior to rupture. The overall fracture surface (Figure 2b) exhibited a rough, fragmented appearance characteristic of brittle fracture. Under higher magnifications (Figure 2c,d), the fracture surface displayed uneven micro-morphology with discrete particulate features, which originated from chlorine-induced oxidative degradation of the HDPE matrix and subsequent brittle crack propagation. When the crack increased to a critical length, stress concentration developed markedly at the crack tip owing to local reduction in the effective pipe-wall thickness. The specimen eventually fractured abruptly, accompanied by minor ductile deformation confined to the outer surface. Such fracture characteristics confirm that exposure to high-temperature chlorinated water promotes the failure-mode transition of HDPE from ductile towards brittle behavior.

The SEM morphologies of HDPE pipe specimens without and after the chlorine-resistance test (sampled away from the brittle crack) are shown in Figure 3. For the sample without the chlorine-resistance test, the low-magnification image (Figure 3a) showed a relatively homogeneous inner surface with only minor scattered particulate impurities, and no obvious crack features were observed. The high-magnification micrograph (Figure 3b) revealed the inherent textured morphology of extruded HDPE, where the polymer matrix remained intact without microcracking or severe matrix degradation. After aging in pressurized chlorinated seawater at 90 °C, at low magnification (Figure 3c), a population of axially oriented surface microcracks were uniformly distributed across inner wall surface. These axially aligned microcracks arose from the hoop stress which was twice the magnitude of axial stress under internal pressure, coupled with an oxidative attack from the chlorinated aqueous environment. Such surface-initiated microcracks originated from the heterogeneous oxidative degradation triggered by chlorinated aqueous environment, which was a typical stress-oxidative cracking feature for HDPE materials. The high-magnification micrograph (Figure 3d) further revealed the microstructural evolution adjacent to crack sites. The HDPE matrix exhibited a wrinkled and undulating texture, accompanied by the emergence of localized fine secondary cracks. The development of surface microcracks and inhomogeneous oxidized domains demonstrates that long-term chlorine exposure induces chemical degradation of HDPE, impairing the structural integrity of the pipe inner surface.

SEM and EDS line scanning were performed across the cross-sectional wall thickness of the HDPE pipe without and after the chlorine-resistance test to characterize the penetration behavior of chloride ions (Cl^−^), as shown in Figure 4. For the sample without the chlorine-resistance test, the low-magnification micrograph (Figure 4a) showed a relatively smooth and intact cross-section, while the high-magnification view (Figure 4b) revealed oriented lamellar structures originating from pipe extrusion, with no obvious microcracks or degradation features observed on the cross-sectional surface. After long-term exposure to pressurized chlorinated seawater at 90 °C, the cross-sectional morphology of specimen 4 (sampled from the region remote from the brittle crack) is shown in Figure 4c,d. The low-magnification image (Figure 4c) still maintained an integral pipe-wall cross-section. At high magnification (Figure 4d), noticeable interfacial separation between oriented lamellae and localized microcracks were visible. Compared with the unaged sample, the aged cross-section displayed a rougher microstructure.

The EDS line-scan profiles (Figure 4e) demonstrated that Cl^−^ remained consistently low along the entire scanned distance up to 2000 μm, indicating no obvious Cl^−^ enrichment within the HDPE bulk matrix. Note that the scanning path was oriented perpendicularly from the inner surface towards the outer surface, as shown in Figure 4c. The carbon signal exhibited a relatively stable intensity across the pipe wall, corresponding to the inherent polymeric composition of HDPE. The weak gold (Au) signal originated from the gold sputtering coating applied for SEM imaging. These elemental distribution results indicated that Cl^−^ could not deeply diffuse and accumulate within the HDPE pipe wall under the tested chlorine exposure condition. Therefore, the brittle failure occurring during the chlorine-resistance test is primarily governed by chlorine-induced surface oxidative degradation, rather than bulk permeation of Cl^−^ or residual chlorine. Although HDPE possesses excellent intrinsic resistance to chloride corrosion, it remains vulnerable to chlorine-initiated surface oxidative deterioration. In addition, the line-scan results in Figure 4e verify that the distinct microstructural differences between the samples without and after the chlorine-resistance test (Figure 4b,d) originate from thermal aging rather than chlorine oxidation.

The test results of failure time *t*_f_ are summarized in Table 2, where the hoop stress *S* is calculated as follows:

(1) $$S=0.5P\left(\frac{D_{0}}{t}-1\right)$$

where *P* is the applied inner pressure, *D*_0_ is the average outside diameter, and *t* is the minimum wall thickness. Table 2 indicated that when the hoop stress decreased 18% ((4.00 − 3.30) × 100%/4.00 = 18%), the failure time *t*_f_ was only increased 6% ((2263 − 2125) × 100%/2263 = 6%). This indicates that there is no linear relationship between the values of *S* and *t*_f_.

Engineering stress–strain curves are shown in Figure 5 for the HDPE pipe specimen without and after the chlorine-resistance test. The tensile test specimen after the chlorine-resistance test was machined from specimen 4 at a location remote from the fracture surface. The tensile test specimen without the chlorine-resistance test exhibited a typical ductile tensile behavior: after yielding at 21.74 MPa under an engineering strain of 12.88%, the material undergoes extensive cold drawing, followed by strain hardening until rupture at a strain approaching 1000%. The post-fracture appearance of this specimen is shown in Figure 6a. It exhibited characteristic ductile failure, with pronounced necking and large-scale plastic stretching within the gauge length. A long, thin drawn-fibril zone formed upon rupture, reflecting the high intrinsic tensile ductility of pristine HDPE.

In comparison, the specimen after the chlorine-resistance test showed significantly deteriorated ductility. Its yield stress dropped slightly to 19.34 MPa at 11.21% strain, and the specimen fractured shortly after yielding without obvious cold-drawing or strain-hardening stages. Residual chlorine oxidation induced microcracks on the inner surface of HDPE pipes, as shown in Figure 3c,d. These microcracks not only reduced the effective cross-sectional area of the specimen but also caused stress concentration, leading to a slight decrease in the yield strength for the specimen after the chlorine-resistance test. Figure 4e indicated that Cl^−^ or residual chlorine could not penetrate into the inner wall of the HDPE pipe; therefore, this ductile-to-brittle failure transition was not caused by residual chlorine oxidation. Comparison between Figure 4b,d revealed that although Cl^−^ could not penetrate into the inner wall of the HDPE pipe, long-term exposure at 90 °C triggered thermal aging of the HDPE pipe. Accordingly, the ductile-to-brittle failure transition arose from thermal aging. The failed specimen is shown in Figure 6b. Only limited local necking was observed near the fracture position, and the length of the stretched fibrils was significantly shortened compared with the specimen without the chlorine-resistance test. This morphological difference demonstrated that chlorine-induced oxidative degradation impaired the plastic deformation capacity of HDPE, reduced its tensile ductility, and shifted the fracture mode toward a less ductile state. Therefore, chlorine oxidation exerts only a minor influence on yield strength, whereas it causes substantial loss of tensile ductility.

## 4. Conclusions

In this paper, the chlorine-resistance behavior of HDPE pipes under pressurized chlorinated seawater at 90 °C was investigated according to the ASTM F2263 standard [17]. The main conclusions are drawn as follows:

(1) Under the combined action of internal hoop stress and chlorinated seawater oxidation, HDPE pipe specimens ultimately exhibited brittle failure after more than 2000 h. No evident macroscopic plastic deformation occurred before final rupture. Numerous axially oriented microcracks nucleated on the pipe inner surface, representing a typical signature of stress-oxidative cracking for HDPE.
(2) An EDS line scan across the pipe-wall thickness indicated that chloride ions exhibited no detectable penetration or accumulation within the bulk HDPE matrix under the tested conditions. Chlorine-induced degradation was therefore a surface-dominated process instead of degradation driven by bulk-phase ion permeation.
(3) Tensile test results confirmed that chlorine oxidation significantly impaired the ductility of HDPE, while the influence on yield strength was relatively limited. Accordingly, the fracture mode transitioned from highly ductile to quasi-brittle.
(4) Within the tested stress range, an 18% reduction in hoop stress only yielded a 6% increase in failure time. Therefore, no obvious linear correlation existed between hoop stress and failure time under chemo-mechanically coupled aging induced by chlorinated seawater.

The present work demonstrates that the service failure of HDPE pipes in chlorinated seawater originates from surface-localized chemo-mechanical degradation. For material selection of HDPE piping intended for seawater transportation, particular emphasis should be placed on surface anti-oxidation performance. To develop chlorine-resistant HDPE pipes, high-performance antioxidants with improved chlorine tolerance need to be explored.

## Figures and Tables

**Figure 1:**
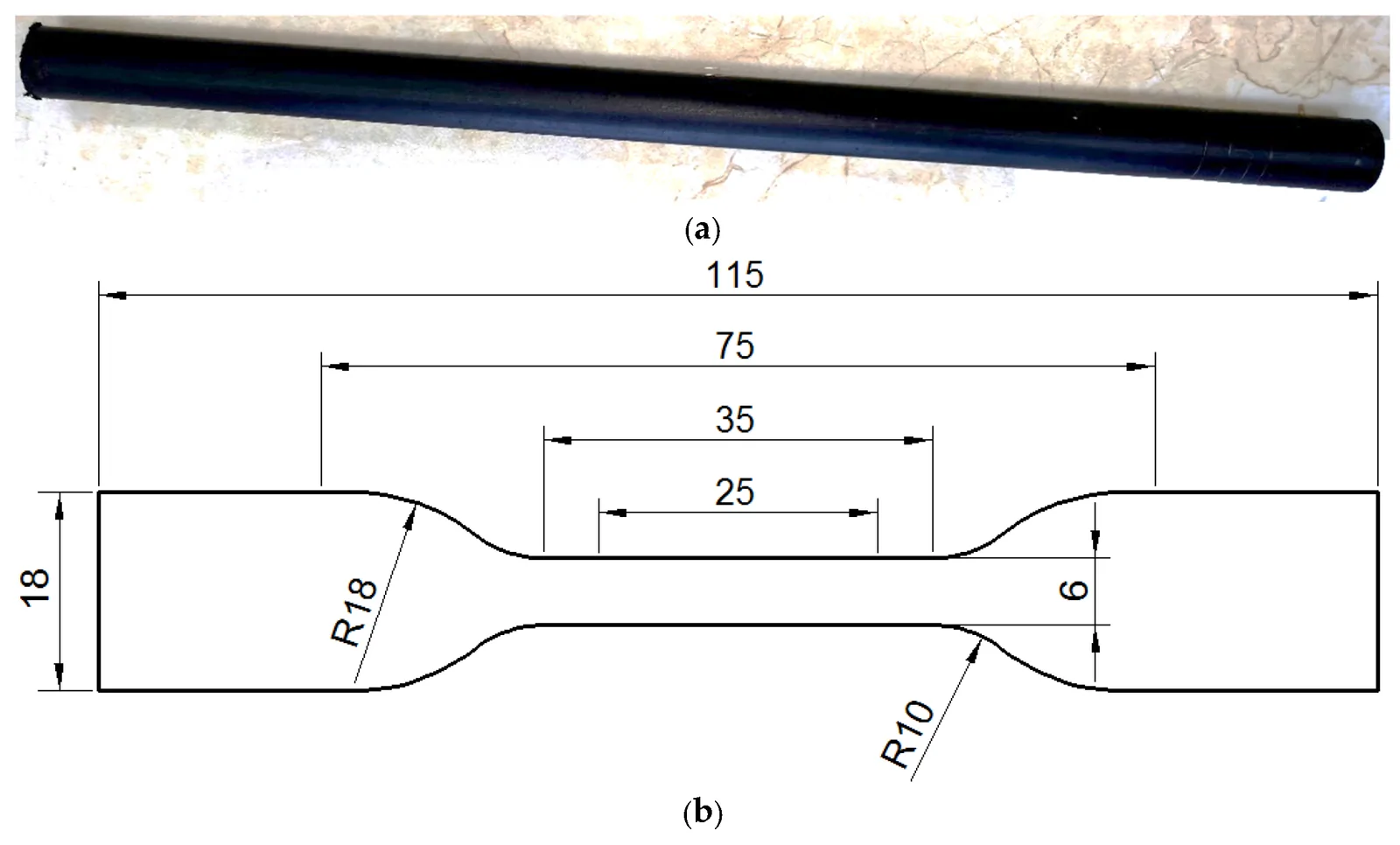

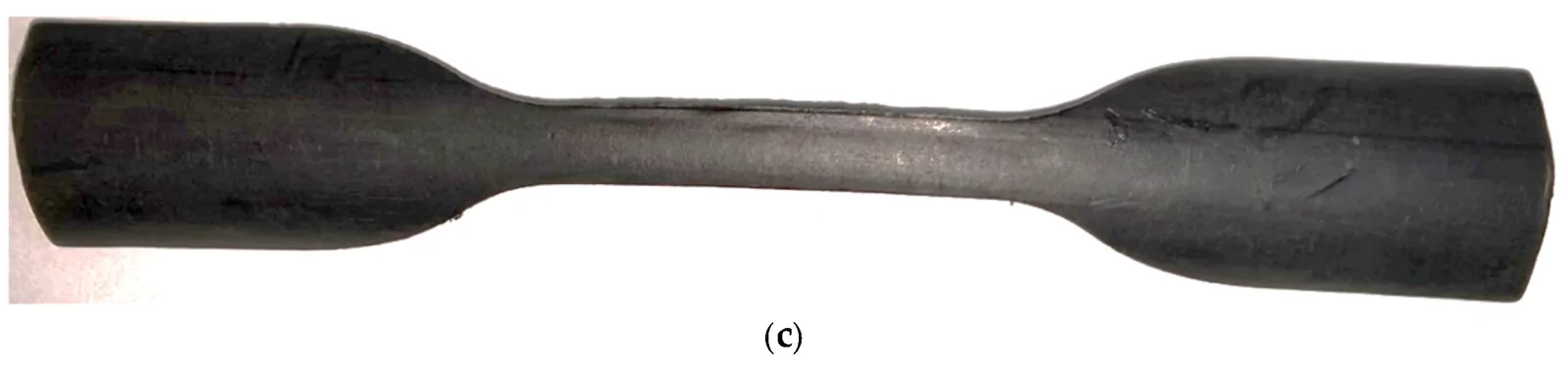
Chlorine-resistance test and tensile test specimens: (**a**) chlorine-resistance test specimen (1/2 in. HDPE pipe with a total length of 400 mm); (**b**) dimensions of tensile test specimen; (**c**) picture of tensile test specimen.

**Figure 2:**
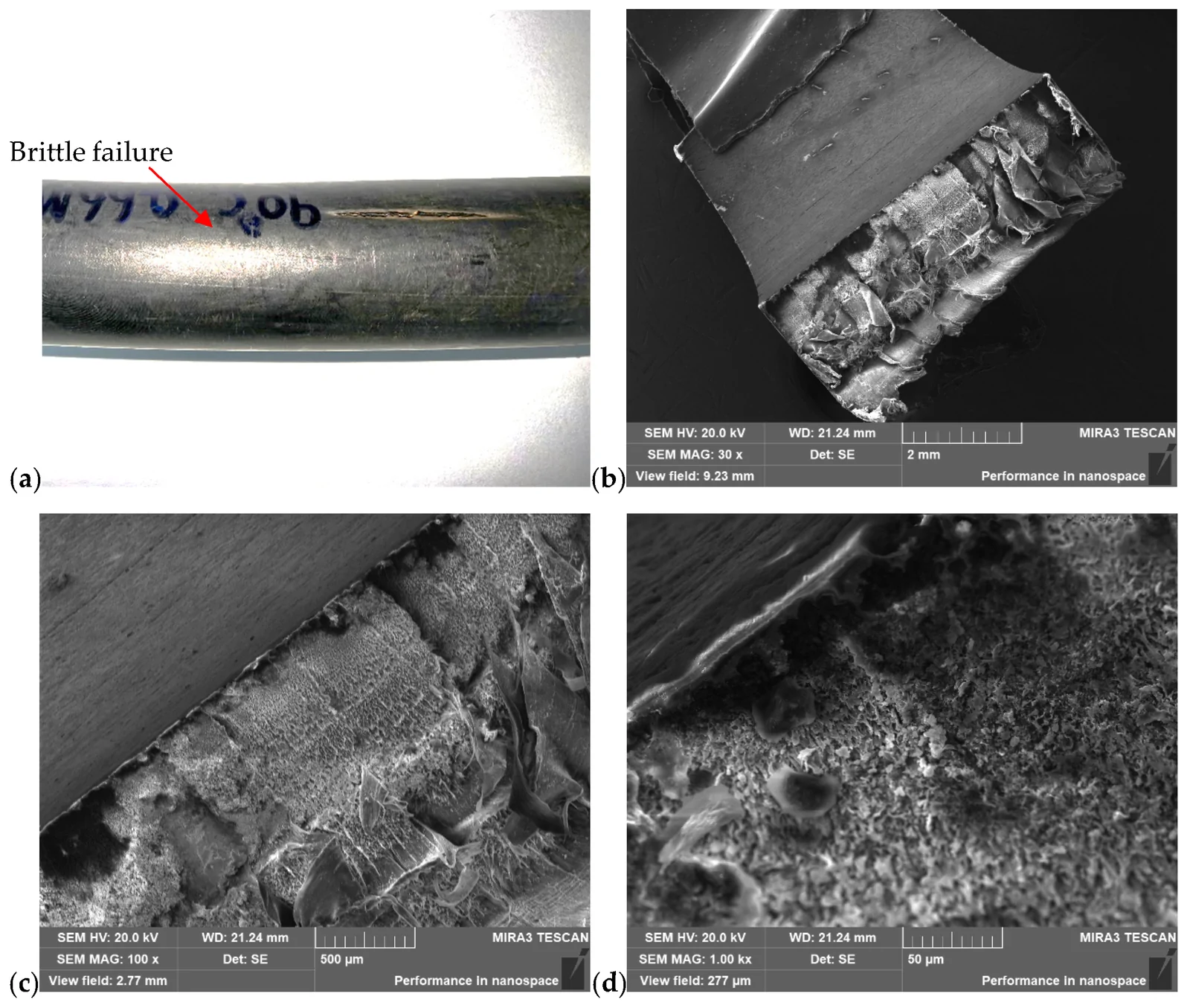
Typical brittle failure of HDPE pipe specimen after chlorine-resistance test at 90 °C: (**a**) macroscopic view of specimen 4 showing the brittle crack; (**b**) low-magnification SEM micrograph of the overall fracture surface; (**c**,**d**) high-magnification SEM images revealing the microstructural features on the fracture surface.

**Figure 3:**
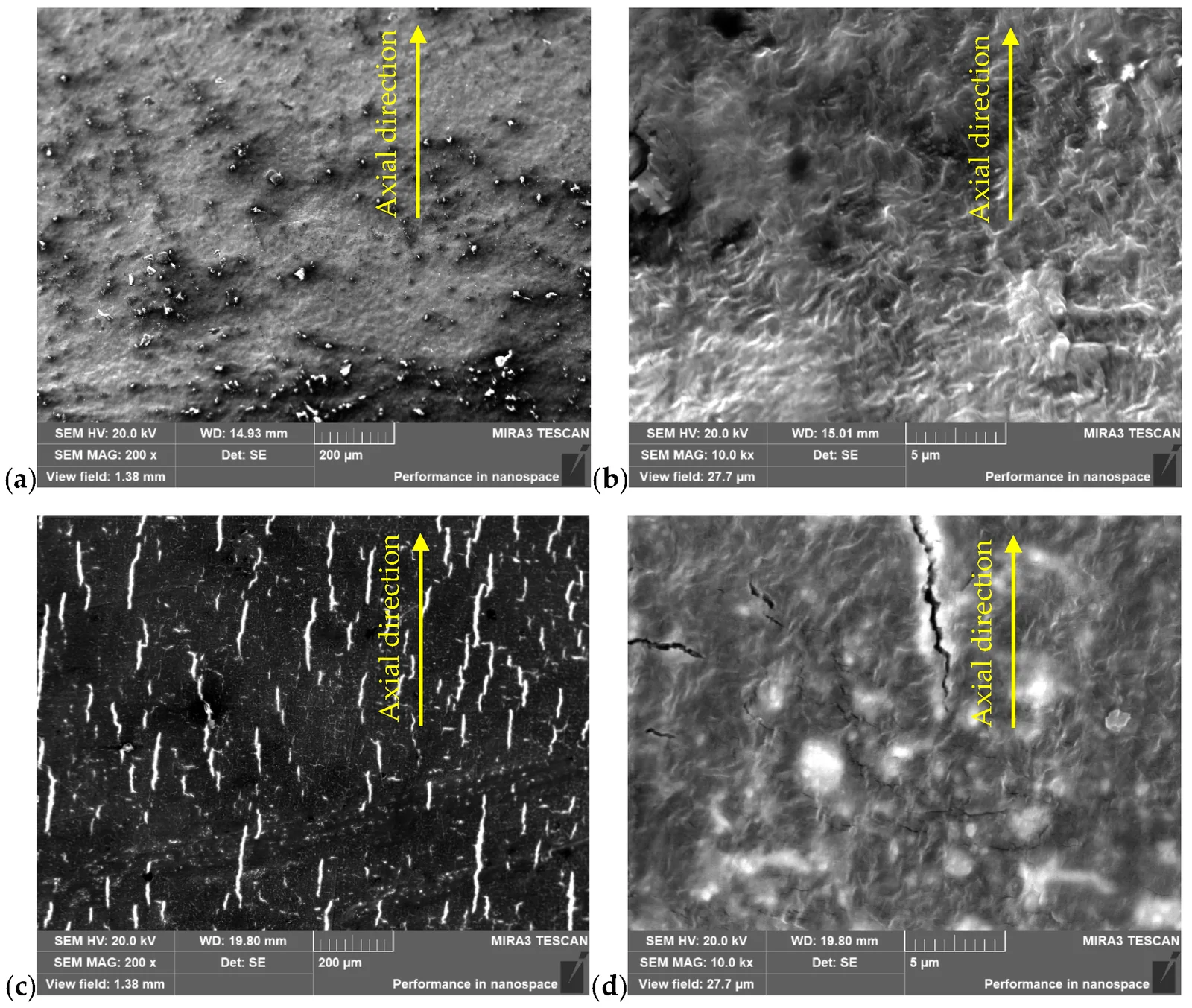
SEM micrographs of inner surface of HDPE pipe: (**a**,**b**) low- and high-magnification images of the sample without chlorine-resistance test; (**c**,**d**) low- and high-magnification images of specimen 4 sampled from the region away from the brittle crack after chlorine-resistance test.

**Figure 4:**
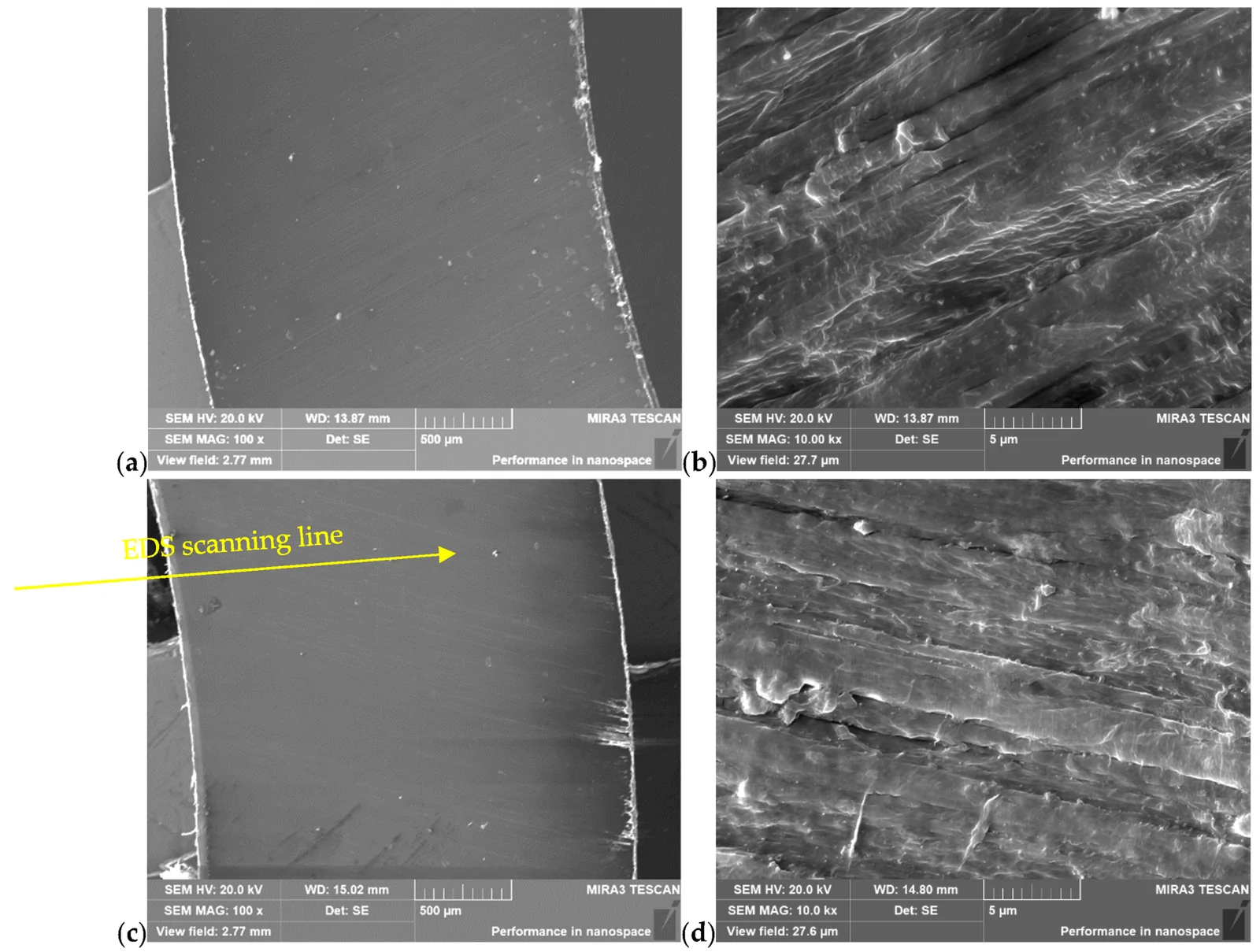

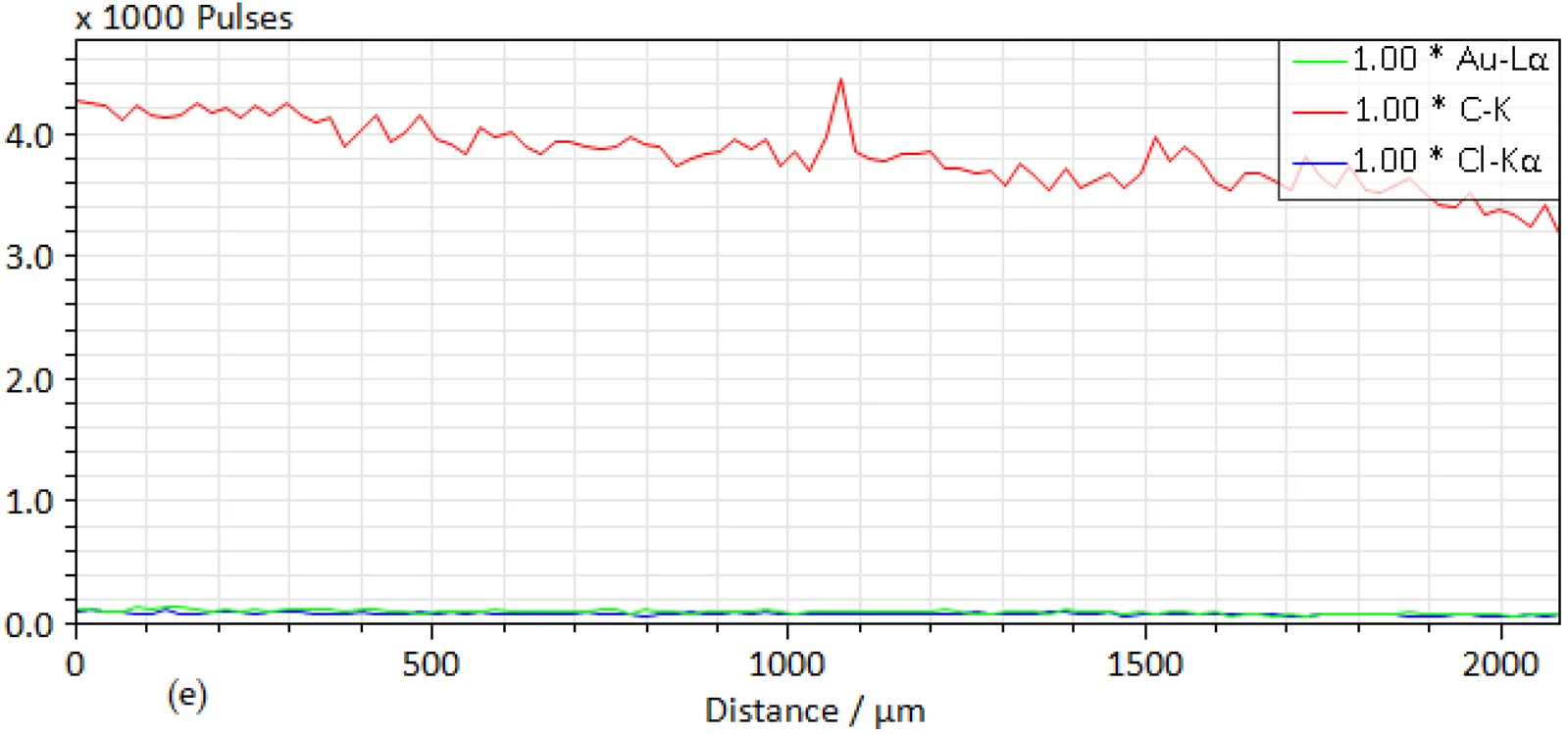
SEM micrographs and distribution of Cl^−^ along the HDPE pipe cross-section: (**a**,**b**) low- and high-magnification images of the sample without chlorine-resistance test; (**c**,**d**) low- and high-magnification images of specimen 4 sampled from the region away from the brittle crack after chlorine-resistance test; (**e**) corresponding EDS line-scan profiles of C, Au, and Cl elements obtained along the scanning path marked in (**c**).

**Figure 5:**
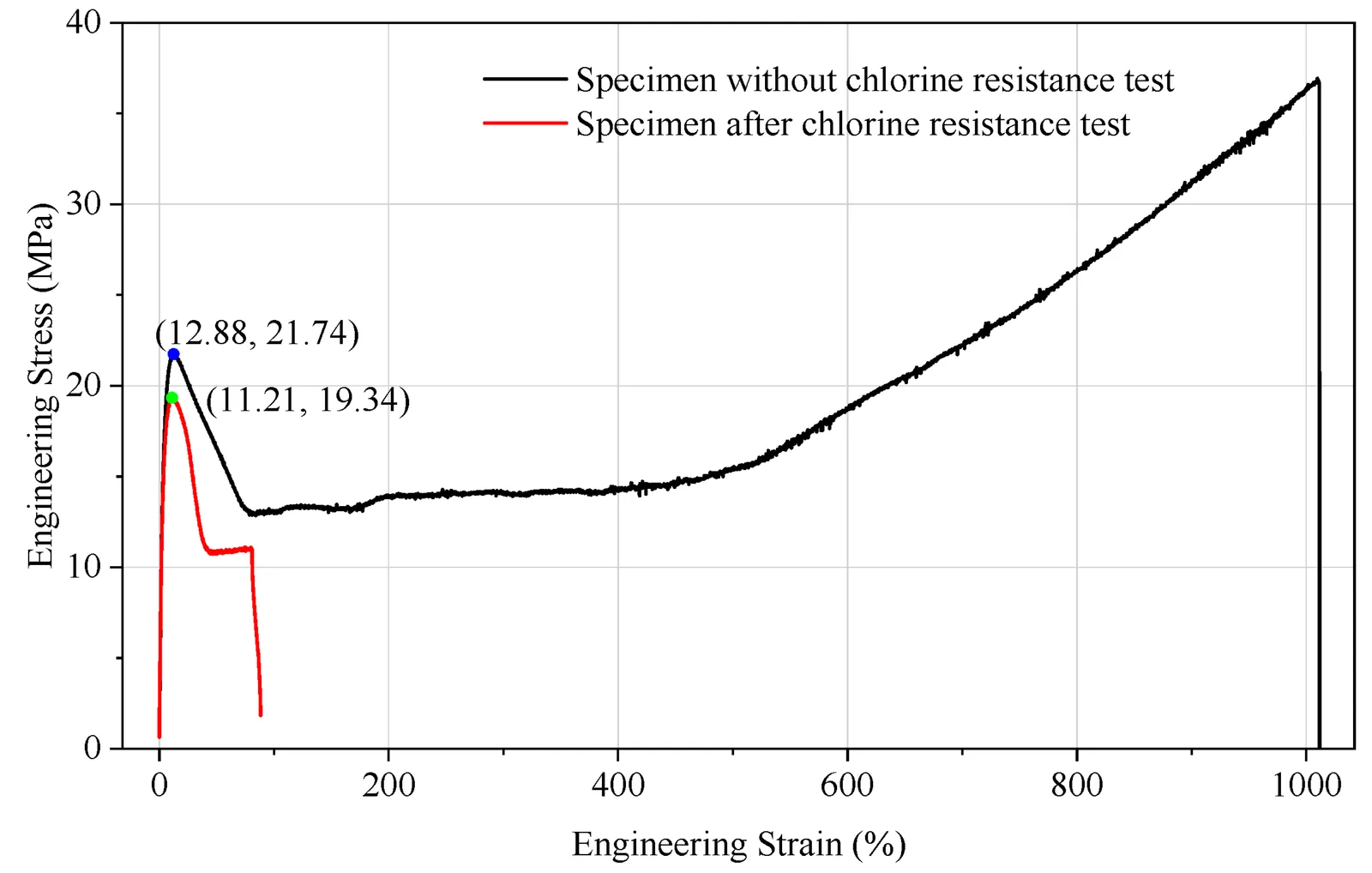
Engineering stress–strain curves of HDPE pipe without and after chlorine-resistance test.

**Figure 6:**
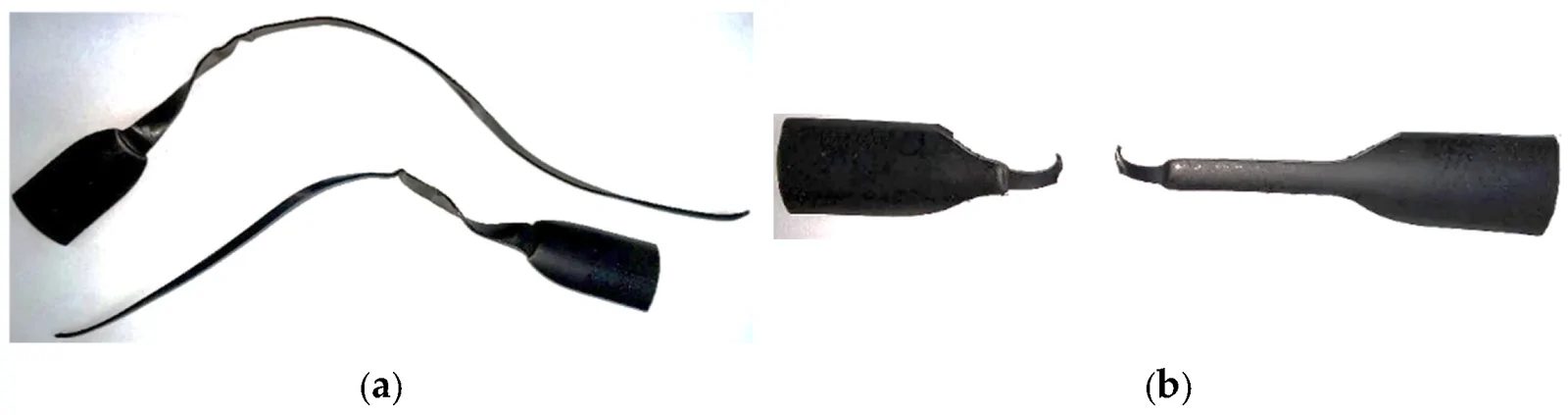
Failed tensile specimens of HDPE pipe: (**a**) specimen without chlorine-resistance test; (**b**) specimen after chlorine-resistance test.

**Table 1:** Composition of experimental seawater.

Cl^−^ (g/L)	SO_4_^2−^ (g/L)	Br^−^ (mg/L)	NO_3_^−^ (mg/L)	Na^+^ (g/L)	K^+^ (mg/L)	Mg^2+^ (mg/L)	Ca^2+^ (mg/L)
9.25	1.44	35.0	<5.0	5.22	199	680	277

**Table 2:** Chlorine-resistance test results of HDPE pipe using seawater with temperature of 90 °C, pH = 6.9, and ORP = 612 mv, and free-chlorine concentration = 0.2 ppm.

Unit	Specimen	Inner Pressure, *P* (MPa)	Hoop Stress, *S* (MPa)	Failure Time, *t*_f_ (h)	Average *t*_f_ (h)
1	1	0.8	4.00	2055	2125
	2			2194	
2	3	0.66	3.30	2261	2263
	4			2264	

## Data Availability

The original contributions presented in this study are included in the article. Further inquiries can be directed to the corresponding authors.
